# Evaluation of the Extraction of Bioactive Compounds and the Saccharification of Cellulose as a Route for the Valorization of Spent Mushroom Substrate

**DOI:** 10.3390/molecules28135140

**Published:** 2023-06-30

**Authors:** Sarah J. Klausen, Anne Bergljot Falck-Ytter, Knut Olav Strætkvern, Carlos Martin

**Affiliations:** 1 Department of Biotechnology, Inland Norway University of Applied Sciences, N-2317 Hamar, Norway; sa.schnell@gmail.com (S.J.K.); anne.falckytter@inn.no (A.B.F.-Y.); knut.stratkvern@inn.no (K.O.S.); 2 Department of Chemistry, Umeå University, SE-901 87 Umeå, Sweden

**Keywords:** spent mushroom substrate, *Pleurotus ostreatus*, bioactive compounds, ultrasound-assisted extraction, subcritical-water extraction, enzymatic saccharification, cellulose

## Abstract

The extraction of bioactive compounds and cellulose saccharification are potential directions for the valorization of spent mushroom substrate (SMS). Therefore, investigating the suitability of different extraction methods for recovering bioactive compounds from SMS and how the extraction affects the enzymatic saccharification is of uppermost relevance. In this work, bioactive compounds were extracted from *Pleurotus* spp. SMS using four extraction methods. For Soxhlet extraction (SoE), a 40:60 ethanol/water mixture gave the highest extraction efficiency (EE) (69.9–71.1%) among the seven solvent systems assayed. Reflux extraction with 40:60 ethanol/water increased the extraction yield and EE compared to SoE. A shorter reflux time yielded a higher extraction of carbohydrates than SoE, while a longer time was more effective for extracting phenolics. The extracts from 240 min of reflux had comparable antioxidant activity (0.3–0.5 mM GAE) with that achieved for SoE. Ultrasound-assisted extraction (UAE) at 65 °C for 60 min allowed an EE (~82%) higher than that achieved by either reflux for up to 150 min or SoE. Subcritical water extraction (SWE) at 150 °C resulted in the best extraction parameters among all the tested methods. Vanillic acid and chlorogenic acid were the primary phenolic acids identified in the extracts. A good correlation between the concentration of caffeic acid and the antioxidant activity of the extracts was found. Saccharification tests revealed an enhancement of the enzymatic digestibility of SMS cellulose after the extraction of bioactive compounds. The findings of this initial study provide indications on new research directions for maximizing the recovery of bioactive compounds and fermentable sugars from SMS.

## 1. Introduction

Edible mushrooms are climate-smart protein-rich food sources with the potential to partially substitute meat, whose production has a high climate impact [1]. Due to the high content of bioactive compounds in mushrooms, their consumption exerts benefits on human health. Mushroom production is a fast-expanding industrial activity involving the cultivation of more than fifty fungal species [2]. *Pleurotus* spp. mushrooms are among the most commercialized in the world market [3].

The generation of crop residues is continuously increasing due to the agricultural expansion driven by global population growth. Disposal by burning is common practice for managing the accumulation of plant residues [4]. However, this practice is against sustainability principles and results in a waste of bioresources that are valuable for bioconversion into products of high economic and social value [5]. Since mushrooms are cultivated on substrates based on plant biomass, using crop and forest residues and by-products from food and wood processing as substrates for mushroom cultivation is a rational bioconversion alternative contributing to sustainable agriculture and forestry.

Mushroom cultivation is directed to produce fruitbodies of edible fungi. After harvesting the fruitbodies, an exhausted residual substrate, i.e., spent mushroom substrate (SMS), is generated. SMS is the main by-product of the mushroom-producing business. Depending on the substrate formulation, the fungal species, and the production system, between three and five kilograms of SMS are formed per kg of cultivated mushrooms [6]. The amount of SMS generated globally by the mushroom industry is expected to reach around 100 million tons by 2026 [7].

SMS is currently regarded as a residue with little value, and its accumulation challenges mushroom producers. Transporting SMS, a bulky material with high moisture content, to disposal sites is expensive, while drying it at the mushroom farm is technically demanding and energy-intensive. Additionally, SMS accumulation is of high environmental concern due to foul odors, greenhouse gases emission from spontaneous anaerobic digestion, and leachate drainage to water sources [8].

SMS valorization following a circular-economy model is critical for the sustainability of the mushroom industry. A valorization option to be considered is based on the potential of SMS as a source of bioactive compounds and polysaccharides [9]. SMS contains bioactive molecules originating from different sources, e.g., (i) the fungal mycelium; (ii) substances secreted by fungal growth; (iii) lignocellulose phytochemicals; and (iv) products of partial degradation of polysaccharides and lignin. Possible bioactive compounds in SMS include polyphenols, polysaccharides, sterols, proteins, vitamins, and other substances.

Recovering bioactive compounds and using them as raw materials for developing novel high-added value products is a promising direction for valorizing SMS. However, while the extraction of bioactive molecules from fungal fruitbodies has been broadly investigated [10], the study of bioactive compounds available in SMS is an entirely new research field. Consequently, the literature has not yet covered essential knowledge on extracting bioactive compounds from SMS.

Bioactive compounds can be recovered from biomass materials using conventional techniques, such as Soxhlet or reflux extraction, performed with various solvents at their boiling temperatures. However, those techniques are time- and energy-consuming, can affect the properties of thermosensitive compounds, and the used organic solvents often present safety, environmental, and toxicological risks. To face that challenge, green-extraction intensification techniques, e.g., ultrasonic assistance, microwaves, or pressurized extractions, with lower energy consumption, shorter extraction times, and non-pollutant solvents, can be used [11].

Saccharification of the polysaccharides contained in the solid residue remaining after extraction of the bioactive compounds and using the resulting sugars in microbial fermentations is another direction for valorizing SMS. We have previously shown that cellulose contained in the SMS of shiitake mushroom (*Lentinula edodes*) is highly susceptible to enzymatic saccharification [12] and that the produced hydrolysates are readily fermentable by baker’s yeast [13]. However, there are no systematic studies on the enzymatic saccharification of SMS of *Pleurotus* spp. mushrooms, and it is unknown how the removal of bioactive compounds by different extraction techniques can affect cellulose saccharification. The main novelty of this work is that bioactive compounds are extracted from *Pleurotus* spp. SMS and that the enzymatic saccharification of the extraction residues is evaluated.

In the current work, the recovery of bioactive compounds from SMS of *Pleurotus* spp. mushrooms by conventional methods, e.g., Soxhlet and reflux extraction, and by green-extraction techniques, e.g., ultrasound-assisted extraction and subcritical water extraction, were investigated. The composition and antioxidant activity of the produced extracts was evaluated using standard methods. Spectrophotometric methods were used to analyze phenolic compounds, carbohydrates, and sterols, and high-performance liquid chromatography (HPLC) was used to identify individual compounds. The enzymatic digestibility of the raw SMS and the extraction residues was assessed using an analytical enzymatic saccharification protocol and HPLC.

## 2. Results and Discussion

**Figure 1:**
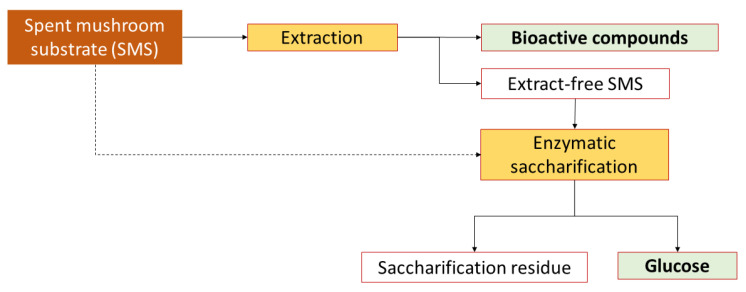
Schematic layout of the experimental procedure followed for the extraction of bioactive compounds from SMS and enzymatic saccharification of the cellulose contained in the extract-free SMS.

This study included the extraction of bioactive compounds from SMS and the enzymatic saccharification of extraction residues (Figure 1). The SMS investigated was generated from cultivating two strains of oyster mushrooms on a hardwood-based substrate. The used strains were a commercial *Pleurotus ostreatus* × *Pleurotus eryngii* hybrid strain known as Black Pearl King Oyster (BPKO) and a wild strain of *P. ostreatus* isolated in Norway, which is hereafter referred to as Norwegian Oyster (NO). The initial substrate contained around 36% (*w*/*w*) cellulose, 28% hemicelluloses, 19% lignin and 9% extractives (Table S1).

### 2.1. Characterization of the Spent Mushroom Substrate

Cellulose was the main constituent of SMS from both fungal strains (Table 1). Cellulose content accounted for 38.3% of the dry mass of BPKO SMS and 37.5% of that of NO SMS. That is slightly higher than the cellulose content in the initial substrate. BPKO SMS also had a higher xylan content (18.5%) than NO SMS (16.6%). The content of anhydroarabinose was comparable for both SMSs. The combined content of xylan and anhydroarabinose in both SMSs was lower than the hemicelluloses’ content of the initial substrate. SMS from BPKO had a lower lignin content (16.0%) than NO SMS (18.4%). For both SMSs, lignin content was lower than in the initial substrate. Extractive content was higher for the SMS of NO (15.0%) than for BPKO SMS (13.5%) and the initial substrate. Water extractives (12.0–13.7% of the SMS dry mass) were predominant over the ethanol extractives (up to 1.5%). The content of water extractives was higher for NO than for BPKO, while that of ethanol extractives was comparable for the SMS from both strains. Carbohydrates dominated the water extractives, while phenolics represented a significant share of the ethanol extractives. Carbohydrates were 72 and 89% of the mass share of the water extractives for NO and BPKO SMS, respectively. Phenolic compounds represented around 20% of the mass share of ethanol extractives. However, it should be noted that a large part of the phenolics had already been solubilized by the water extraction, which was performed before the ethanol extraction. Ergosterol was identified after alkali-assisted ethanol extraction. Ash content was higher for the NO SMS (2.7%) than for the BPKO one (2.0%).

The lower content of lignin and hemicelluloses in the SMSs than in the initial substrate can be attributed to degradation during cultivation. *Pleurotus* spp., like other white-rot fungi, degrade, either partially or entirely, wood structural components and use the degradation products as nutrients [14]. The higher content of extractives in the SMSs than in the initial substrate is also a consequence of the fungal growth. Some extractive compounds originated from the fungal degradation of the lignocellulosic components of the substrate. For example, the phenolic compounds result from lignin degradation by oxidative enzymes, such as peroxidases and laccases, that are secreted by fungi during cultivation [3]. Other extractive compounds originated from fungal biomass. That includes carbohydrates, such as β-glucans [15], and proteins and sterols from the fungal mycelium, which remains in the SMS by the end of cultivation [16].

### 2.2. Extraction of Bioactive Compounds from SMS Using Conventional Methods

The high content of extractive compounds in SMS makes it of interest as a source of bioactive compounds. The extraction of bioactive compounds, provided that appropriate methods are identified, might provide a suitable alternative for resource recovery from SMS [16]. In this work, bioactive compounds were extracted from SMS using both conventional and modern techniques, and the remaining solids were then subjected to enzymatic saccharification (Figure 1).

Conventional methods, including maceration, percolation, and reflux extraction, have been used to extract bioactive compounds from plant-based material for decades [17]. Reflux is more effective than percolation and maceration, but the three methods require a long extraction time using large volumes of organic solvents. Another conventional extraction method is the Soxhlet technique, which is a reflux system with an incorporated siphoning device. It is a more efficient approach since it allows continuous extraction combining reflux and percolation methods. However, it still requires high temperature and long extraction time, which increase the possibilities of thermal degradation.

In this study, Soxhlet extraction with seven solvent systems was performed for SMS from BPKO mushroom. After that, the solvent mixture giving the best results on yield, composition, and antioxidant activity was used in reflux for several extraction times.

#### 2.2.1. Soxhlet Extraction

**Figure 2:**
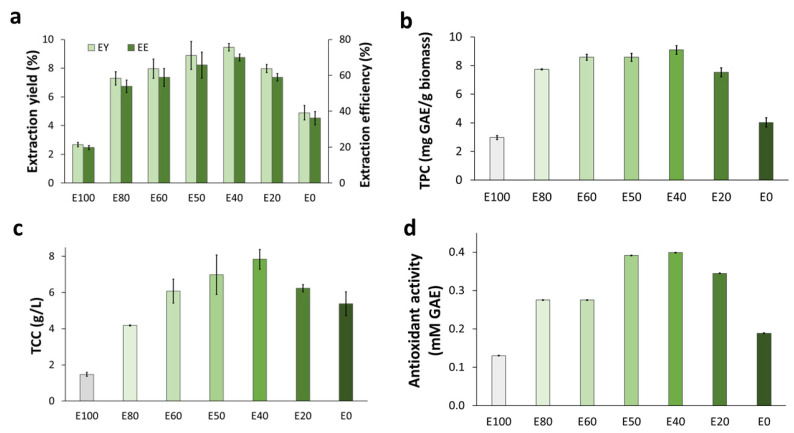
Soxhlet extraction applied to BPKO SMS. The extraction was held for four hours using different ethanol/water mixtures. Extraction yield and extraction efficiency (**a**), recovery of total phenolics, (**b**) concentration of total carbohydrates (**c**), and antioxidant activity (**d**). The numerals in the horizontal axis indicate ethanol (E) volumetric share in the solvent mixture. Mean values from triplicate experiments were used to build the graphs. The error bars show the standard deviations.

In Soxhlet extraction applied to SMS from the BPKO strain, the seven solvent systems resulted in different extraction yields (EYs). The highest EY (9.5% (*w*/*w*)) was achieved with the 40:60 ethanol/water mixture (E40 in Figure 2a), which corresponded to an extraction efficiency of 69.9%. The EY decreased proportionally with the increase in the share of any solvent component in the mixture.

As the content of either water or ethanol increased, the recovery of total phenolic compounds decreased. The highest recovery of total phenolic compounds (9.1 mg GAE/g biomass) in the Soxhlet extracts was detected in the sample from the extraction with a 40:60 ethanol/water mixture (E40), and they were only slightly lower for the E50 and E60 mixtures (Figure 2b). The recovery of phenolic compounds from SMS was higher than that previously reported for the Soxhlet extraction of dehydrated *P. ostreatus* fruitbodies (3.9–6.4 mg GAE/g), but it was lower than that achieved for fresh fruitbodies (14.3–21.9 mg GAE/g) [18]. The recovery of phenolics in the current study was comparable with reported results for other plant-biomass materials using a similar extraction approach, but some particularities were observed. For example, the range of recovery of total phenolics in this work (3.0–9.1 mg GAE/g biomass) was comparable with that achieved by Espinosa et al. [19] by Soxhlet extraction of orange-peel waste with different ethanol/water mixtures (2.43–8.55 mg GAE/g biomass). However, the extractability profile was different in both studies. In Espinosa et al.’s study, the highest recoveries were achieved with solvent systems containing at least 75% (*v*/*v*) ethanol, whereas in the current work, the highest values were reached with mixtures containing between 40 and 60% (*v*/*v*) ethanol. The different behavior can be attributed to the different compositions of the phenolic fraction in both materials. Apparently, more polar compounds are predominant in the phenolic fraction of SMS than in that of orange-peel waste. That is in line with the high share of phenolic compounds found in the water extractives’ fraction during the SMS characterization of the SMSs (Table 1).

The concentration of total carbohydrates was the lowest when only ethanol was used as the solvent, and it increased as the volumetric share of ethanol in the solvent system decreased from 100 to 40% (Figure 2c). The maximum value was reached for the E40 mixture. Then, a decrease was observed for E20 and E0. The increased concentrations in extractions with decreasing ethanol shares in the solvent mixture agree with the existing knowledge on the solubility of carbohydrates in ethanol/water mixtures [20]. The observed decrease in the concentrations in extractions with solvent systems with lower ethanol shares (20 and 0%) might be related to the predominance of polysaccharides or relatively large oligosaccharides, which typically have lower ethanol solubility than that of monosaccharides [21]. The low solubility of SMS carbohydrates in ethanol can be exploited for their isolation from water extracts using ethanol precipitation.

The highest antioxidant activity (0.4 mM GAE) was found in the extract from the E40 solvent mixture (Figure 2d). The lowest value (0.1 mM GAE) was observed when ethanol was the only solvent (E100). Antioxidant activity correlated well with the concentration of total phenolics in the extracts (see Section 3.5).

#### 2.2.2. Reflux Extraction

**Figure 3:**
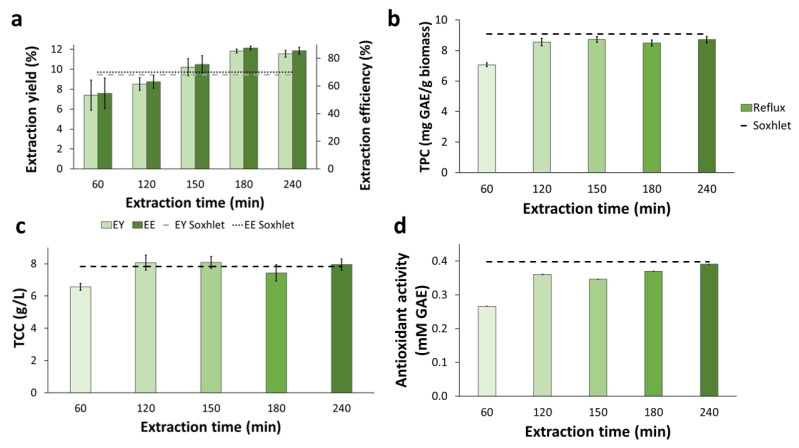
Reflux extraction applied to BPKO SMS. Extraction yield and extraction efficiency (**a**), recovery of total phenolics (**b**), concentration of total carbohydrates, and (**c**) antioxidant activity (**d**). Mean values from triplicate experiments were used to build the graphs. The error bars show the standard deviations.

Reflux extraction applied to BPKO SMS lasted between 60 and 240 min, and it was performed with the E40 solvent system, which gave the best results in the Soxhlet experiment (see Section 2.2.1). The extraction yield and efficiency increased proportionally with the reflux time for the first three hours (Figure 3a). After that, a plateau was reached. Reflux lasting 150 min or longer resulted in higher EY and EE than Soxhlet extractions. For facilitating the comparison, horizontal lines denoting the best results achieved by Soxhlet extraction are included in Figure 3.

The extract from the 60-min reflux had a significantly lower (*p*-value < 0.05) concentration of total phenolic compounds (Figure 3b) and total carbohydrates (Figure 3c) than those achieved with Soxhlet extraction using the same solvent mixture (Figure 2b). For the concentration of phenolic compounds from more extended reflux extractions, the difference was less remarkable, but it was still slightly lower than for Soxhlet extraction. Notably, no significant differences were observed between the recoveries of total phenolics in extracts from reflux extractions lasting 120 min or more, which were all in the range between 7.1 and 8.7 mg GAE/g biomass. For total carbohydrates, the concentrations in all the extracts from reflux lasting 120 min or more were comparable to the highest value achieved with Soxhlet extraction.

The antioxidant activity of all reflux extracts of the BPKO SMS, except for that from the 60-min lasting extraction, showed comparable results (Figure 3d). The antioxidant activity of the 60-min extract (0.3 mM GAE) was significantly lower (*p*-value < 0.05) than the highest value achieved in Soxhlet extraction (0.4 mM GAE).

### 2.3. Extraction of Bioactive Compounds from SMS Using Green Techniques

The conventional extraction methods are usually performed with organic solvents and require a high volume of solvent and a long time, which can affect the properties of the extracted molecules. Green, or non-conventional, extraction methods have features such as lower consumption of organic solvents, shorter extraction times, and higher efficiency and selectivity, which allow overcoming the disadvantages of conventional extraction methods [22]. Two green-extraction methods, namely ultrasound-assisted extraction and subcritical water extraction, were evaluated for SMS in this study.

#### 2.3.1. Ultrasound-Assisted Extraction

UAE under optimized conditions is a valuable green technique for extracting bioactive compounds from plant biomass [23]. In this work, a full-factorial 3^2^ experimental design was used to investigate the effect of temperature and time on the yield and efficiency of UAE of BPKO SMS (Table 2). Since UAE has not been reported before for SMS, the selected operational conditions were based on reported data for other plant-based residual materials [11,19,24,25].

**Figure 4:**
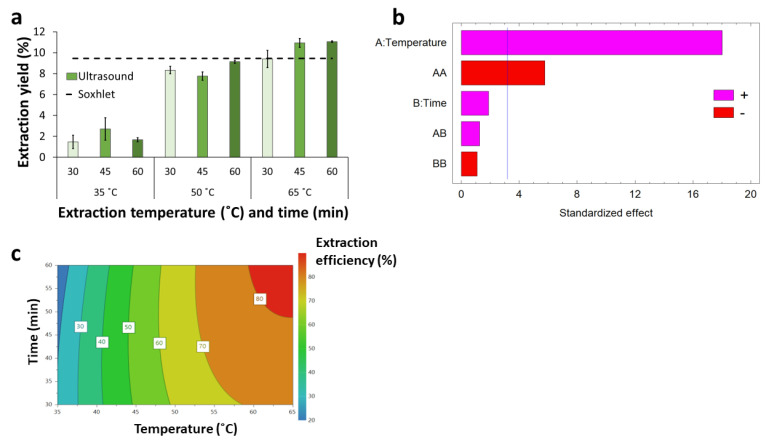
Extraction yield and efficiency (%) for ultrasound-assisted extraction applied to BPKO SMS. Extraction yield (**a**), Pareto chart of standardized effects (**b**), response contour plot (**c**).

Ultrasound-assisted extraction applied to BPKO SMS at 35 °C resulted in comparable responses independently of the extraction time. Increasing the temperature resulted in an increase in the EY from 1.5–2.7% at 35 °C to 9.4–11.1% at 65 °C (Figure 4a). The differences between results from different temperatures were statistically significant (*p*-value < 0.05). For the experiments at 35 and 50 °C, the extraction yield was lower than the highest value achieved with Soxhlet extraction. However, UAE at 65 °C resulted in comparable and higher yields than Soxhlet extraction. This result agrees well with a previous report on orange peel waste, where UAE at 80 °C resulted in higher extraction yield than Soxhlet extraction but compared negatively at either 30 or 60 °C [19]. For UAE at 50 and 65 °C, an increase in the EY with time was observed as a general trend. The highest EY (11.1%) was observed for the extraction at 65 °C for 60 min. The trend observed for the EE was similar to the one observed for the EY (Table 2). For EY and EE in UAE, less time and lower temperature were required to reach comparable results as those obtained by Soxhlet and reflux extractions.

The significance analysis revealed that the temperature was the independent factor exerting the most important estimated effect on the extraction efficiency, as shown by the Pareto chart of standardized effects (Figure 4b). The positive sign indicates that a temperature increase enhances the extraction efficiency. The quadratic term of the temperature also exerted a significant effect but had a negative sign. No significant effects were exerted by the extraction time, its quadratic term, and its interaction with the temperature. Previous reports on longer UAE performed for orange peel waste showed that for that material and under those conditions, time significantly affected extraction yield in the range of 10–60 min but had only a marginal effect in the range of 60–180 min [19].

An empirical model (Equation (1), R^2^ = 97.5%) describing the effect of the operational conditions on extraction efficiency was proposed based on the experimental results. The factors exerting no significant effects were excluded from the model. The model is shown as Equation (1), in which *EE* is the extraction efficiency (in %), and *T* is the temperature (in °C).

(1) $$EE=63.27+62.83\;T-34.77\;T^{2}$$

The response contour plot provides a better description of the estimated effect of the temperature on the extraction efficiency (Figure 4c). The graph shows that the EE increased sharply with temperature increase from 35 °C to around 50 °C. In contrast, further temperature increases exerted a relatively moderate effect. A region with maximal EE can be reached at around 60–65 °C and extraction times above 50 min.

**Figure 5:**
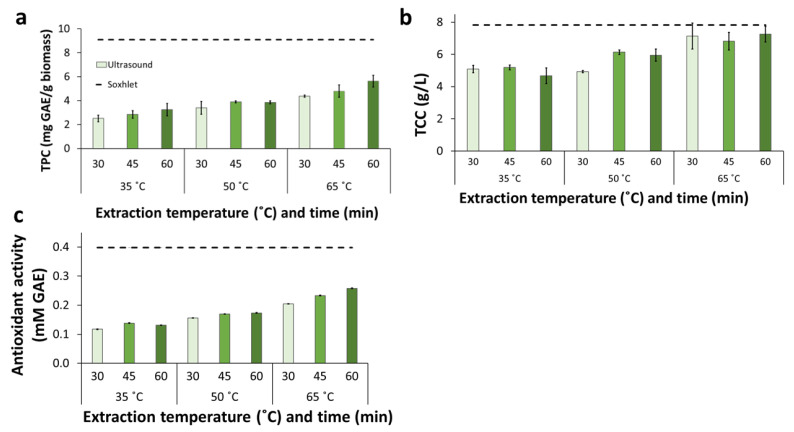
Ultrasound-assisted extraction applied to BPKO SMS. Recovery of total phenolics (**a**), concentration of total carbohydrates (**b**), and antioxidant activity (**c**). The extraction was performed using the E40 solvent mixture. Mean values from triplicate experiments were used to build the graphs. The error bars show the standard deviations.

The recovery of total phenolic compounds ranged from 2.5 mg GAE/g biomass in the extracts from UAE at 35 °C for 30 min to 5.6 mg GAE/g in those from extractions at 65 °C for 60 min (Figure 5a). Those concentrations were significantly lower (*p*-value < 0.05) than the highest values observed for Soxhlet (Figure 2b) and reflux (Figure 3b) extractions. Nonetheless, the concentrations of phenolics achieved in all UAE experiments at 65 °C were higher than those from Soxhlet extractions that used either pure ethanol (E100) or water (E0) as solvents.

For UAE at 35 and 50 °C, the concentrations of total carbohydrates in the extracts were between 4.7 and 6.0 g/L (Figure 5b). That concentration range is clearly lower than the highest values found for Soxhlet (Figure 2c) and reflux (Figure 3c) extractions. There was a general increase in concentration with the increase in temperature, as can be shown by the highest values for 35 °C (5.2 g/L), 50 °C (6.0 g/L), and 65 °C (7.3 g/L). As a rule, extracts from UAE at a given temperature displayed carbohydrate concentrations in a comparable concentration regardless of the extraction time. The highest total carbohydrate concentrations were observed in extracts from experiments at 65 °C (6.8–7.3 g/L). Those values were only slightly lower than the results in the best-performing Soxhlet and reflux extractions.

The antioxidant activity of UAE extracts increased with temperature (Figure 5c). For experiments at 35 and 50 °C, the results were somewhat comparable for different extraction times at a given temperature except for extractions at 65 °C, which showed a general increase with extraction time. In addition, even for the extract from UAE at 65 °C and for 60 min, the antioxidant activity was lower than for Soxhlet and reflux extractions.

#### 2.3.2. Subcritical-Water Extraction

**Figure 6:**
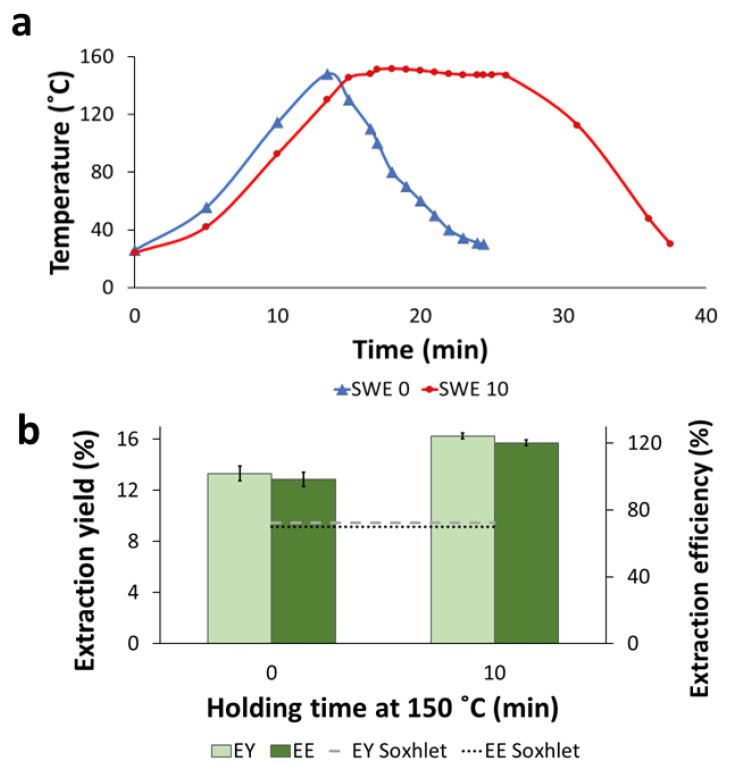
Subcritical-water extraction applied to BPKO SMS. Temperature profiles (**a**), extraction yield, and extraction efficiency (**b**). The extraction was held with either 0 or 10 min holding at 150 °C. Mean values from triplicate experiments were used to build the graph in panel (**b**). The error bars show the standard deviations.

In a final experiment, SWE, e.g., extraction with water in a pressurized reactor, was performed for the BPKO SMS. SWE was performed either non-isothermally (SWE 0) (by heating an SMS suspension followed by cooling it immediately after reaching 150 °C) or with a holding time of 10 min at 150 °C (SWE 10). The temperature profiles of both experiments are shown in Figure 6a. The severity factor (SF) was 1.8 for non-isothermal SWE and 2.3 for the experiment with a holding time of 10 min.

The extraction yield was significantly higher (*p*-value < 0.05) for SWE with 10-min holding at 150 °C (16.3%) than for the non-isothermal experiment (13.3%) (Figure 6b). The same trend was observed for the extraction efficiency. The EY and EE were higher for SWE than the other extraction methods, and the EE of SWE 10 exceeded 100% (*w*/*w*). That phenomenon can be attributed to an increase in the apparent mass of extractives by the partial solubilization of cell wall constituents, mainly hemicelluloses. It is known that the partial solubilization of the hemicelluloses can occur in subcritical water at temperatures around 150 °C, especially if the temperature is held for a certain time [26].

**Figure 7:**
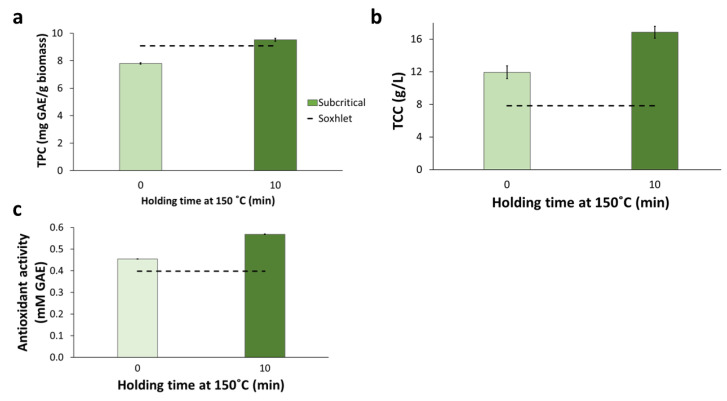
Subcritical-water extraction applied to BPKO SMS. Recovery of total phenolics (**a**), concentration of total carbohydrates (**b**), and antioxidant activity (**c**). The extraction was held with a holding time of either 0 or 10 min at 150 °C. Mean values from triplicate experiments were used to build the graphs. The error bars show the standard deviations.

The recovery of total phenolic compounds (Figure 7a) and the concentration of total carbohydrates (Figure 7b) in the SWE extracts were higher for SWE 10 than SWE 0. For both extracts, the content of phenolics was comparable with the values resulting from Soxhlet and reflux extraction, but it was higher than for UAE. The content of total carbohydrates was significantly higher (*p*-value < 0.05) than for Soxhlet extraction, as illustrated by the position of the horizontal dashed line in Figure 7b. It was also higher than for reflux and UAE. The extract from SWE 10 had the highest concentration of carbohydrates (16.8 g/L) among the extracts from all extraction methods. That high concentration might be linked with the presence of sugars resulting from the degradation of hemicelluloses.

As for other parameters, the antioxidant activity was higher in the extract from the SWE 10 experiment than in the SWE 0 one (Figure 7c), and both extractions displayed higher antioxidant activity than the extracts from Soxhlet, reflux, and ultrasound extractions.

The solvent used in SWE was distilled water, the so-called E0 solvent system in Soxhlet extractions (see Section 2.2.1). When comparing the E0 Soxhlet extraction with both SWE experiments, it is evident that SWE resulted in higher yield, efficiency, total phenolics and carbohydrates concentration, and antioxidant activity than Soxhlet extraction. Furthermore, those results were achieved in a much shorter extraction time. That confirms the potential of subcritical-water extraction as an efficient method for achieving suitable extraction parameters more time-effectively than conventional extraction under atmospheric conditions [27].

### 2.4. Extraction of Bioactive Compounds from SMS of the Wild Strain of P. ostreatus

Soxhlet-, reflux-, and ultrasound-assisted extraction were applied to the NO SMS using the conditions resulting in the best results for the BPKO SMS. A comparison of the results for the extraction of both SMSs is shown in Table 3. The E40 solvent mixture was used in all the experiments.

The extraction yield and extraction efficiency were higher for NO SMS than for BPKO SMS (Table 3). The differences were statistically significant (*p*-value < 0.05) for the EY for all the extraction methods. The EE differences were significant for reflux extraction but not for Soxhlet extraction or UAE.

The recovery of total phenolics in Soxhlet extracts was significantly higher for BPKO SMS than for NO SMS, but in reflux extracts and UAE extracts, it was the other way around (Table 3). The highest recovery of phenolics (9.5 mg GAE/g biomass) was found in the reflux extracts of the NO SMS.

On the other hand, in Soxhlet extracts, the concentration of total carbohydrates was higher for BPKO SMS than for NO SMS. In contrast, the values were comparable for both SMSs in reflux extracts and UAE extracts. The highest concentration of total carbohydrates (8.1 g/L) was found in the reflux extract of the BPKO SMS. The antioxidant activity of the extracts from all the extraction methods was significantly higher (*p*-value < 0.05) for NO SMS than for BPKO SMS (Table 3).

The higher EY, EE, concentration of total phenolic compounds, and antioxidant activity observed in the extracts of the SMS of the wild strain are in accordance with the content of extractive compounds in the SMS (Table 1). Furthermore, these results might also be interpreted as an indication that NO SMS has a higher susceptibility to being extracted and to yielding extractive compounds with higher antioxidant activity than BPKO SMS.

### 2.5. Phenolic Acids in the Extracts and Their Correlation with the Antioxidant Activity

**Figure 8:**
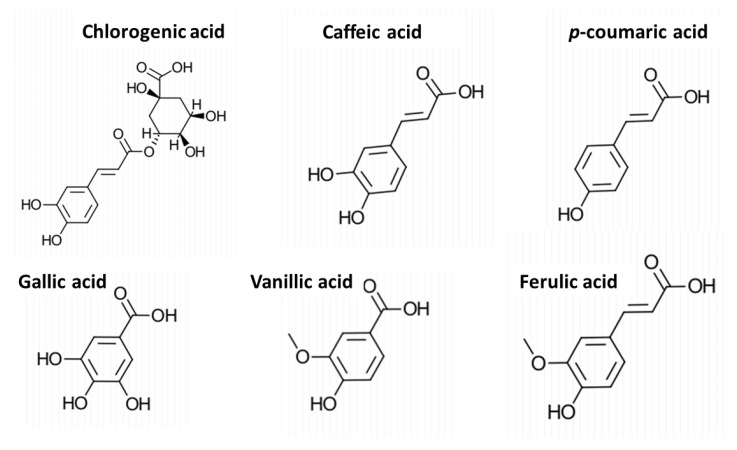
Phenolic acids identified in extracts of BPKO SMS.

Six phenolic acids were identified in the extracts obtained by different methods (Figure 8). Vanillic, gallic, and chlorogenic acids were the compounds detected in the highest concentrations in most of the extracts (Table 4). The concentrations of 2,3-dihydrobenxoic acid and protocatechuic acid were below the detection limits. The highest concentrations of vanillic acid (16.4 mg/L) and gallic acid (11.1 mg/L) were found in the SWE extract, while the highest concentration of chlorogenic acid (7.3 g/L) was detected in the Soxhlet extract using the E40 solvent mixture.

The Soxhlet E40 extract also had the highest concentrations of *p*-coumaric acid (2.3 mg/L) and ferulic acid (1.1 mg/L) (Table 4). For Soxhlet extraction, the concentration of total identified phenolic acids and the concentrations of gallic and vanillic acids increased with the increase in ethanol share in the solvent mixture. Gallic acid concentration increased from 3.0 mg/L in the E20 extract to 5.5 g/L in the E80 extract, while vanillic acid increased from 6.9 to 12.4 mg/L. This is an expected result, as gallic and vanillic acids are more soluble in ethanol than water [28,29]. For other compounds, no clear trend was observed. For caffeic acid (0.6–0.7 mg/L), ferulic acid (0.8–1.1 mg/L), and *p*-coumaric acid (1.6–2.3 mg/L), the concentrations remained comparable for all the extracts.

For reflux extraction, regardless of the extraction time, comparable concentrations were observed for each identified phenolic acid (Table 4). The exception was vanillic acid, which showed increased concentration with extended extraction time. The phenolic acid displaying the highest concentration in any reflux extract was vanillic acid (5.8 mg/L in the 240-min extract). The concentrations found in reflux extracts were generally comparable to those from Soxhlet extractions with the E20 solvent mixture.

For UAE, a general increase in the concentration of most phenolic acids was observed with the increase in temperature and time (Table 4). The exceptions were gallic acid, whose highest concentration (3.2 mg/L) was observed for the extraction at 50 °C and 45 min, and vanillic acid, which did not show a clear trend. The highest concentration for any of the identified compounds in UAE was found for chlorogenic acid (4.6 mg/L) in the extract from the experiment at 65 °C and 60 min. UAE yielded lower concentrations for all the phenolic acids than those observed for the E40 Soxhlet extraction and reflux experiments. The most remarkable case was caffeic acid, whose concentration in UAE extracts corresponded to only around half the value in the reflux extracts and one-fourth in Soxhlet extracts. That phenomenon can be attributed to the degradation of caffeic acid under ultrasound treatment, as shown previously in a study of sonication of phenolic acids in a model system [30]. The highest combined concentration of all identified phenolic acids (36.5 mg/L) was found for SWE (Table 4). The sum of the phenolic acids identified in the Soxhlet, reflux, and UAE extracts was within the range of, respectively, 17–27.8, 14.0–16.1, and 10.6–13.5 mg/L. SWE also resulted in the highest concentrations of several of the individual compounds, e.g., caffeic acid (1.7 mg/L), gallic acid (11.1 mg/L), and vanillic acid (16.4 mg/L).

Some phenolic acids have been reported before in fruitbodies of edible mushrooms [31]. The content of each phenolic acid identified in this work was lower than those previously reported for extracts *P. ostreatus* fruitbodies [32]. The partial identification of phenolic acids in SMS has been reported for shiitake mushrooms [33]. However, to our knowledge, there are no previous studies on identifying phenolic acids extracted from the SMS of oyster mushrooms.

**Figure 9:**
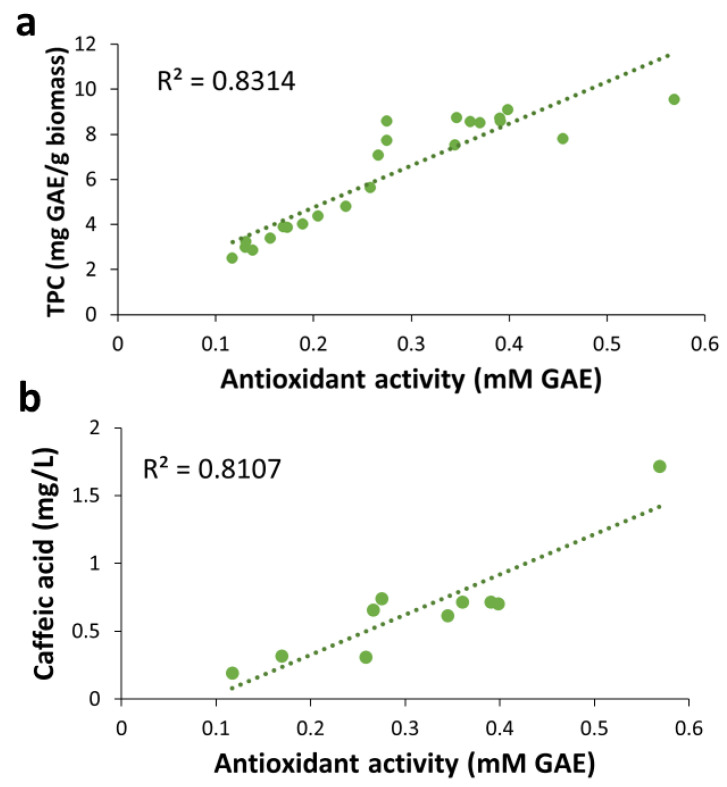
Scatter plots of the antioxidant activity in extracts from BPKO SMS versus concentrations of total phenolics (**a**) and caffeic acid (**b**). Samples are from different extraction methods.

The concentrations of total phenolic compounds, individual phenolic acids, and total carbohydrates in all the extracts were correlated to the corresponding antioxidant activity, which was expressed as ferric-ion reducing antioxidant power (FRAP). A strong, significant, and positive correlation between the concentration of total phenolic compounds (mg/L) and the ferric-reducing power (mM GAE) (R^2^ = 0.80, Pearson correlation coefficient (PCC): 0.89, *p*-value < 0.05) was revealed (Figure 9a). That is an indication of a strong contribution of the total phenolic compounds to the observed antioxidant activity in the samples. This result agrees with previous studies reporting correlations between antioxidant activity, using various criteria, and total phenolic content (TPC) in extracts of fungal biomass and other materials. For example, Martínez-Flores et al. [18] found a strong positive correlation between TPC and antioxidant activity, determined by both the radical scavenging activity (DPPH) and the 2,2′-azino-bis(3-ethylbenzothiazoline-6-sulfonic acid) (ABTS) assay, in aqueous ethanol extracts of *P. ostreatus* and *Pleurotus djamor* fruitbodies. Yim et al. [34] reported a strong significant correlation in aqueous extracts of *P. ostreatus* fruitbodies using the FRAP assay. For other biomass materials, Showkat et al. [35] reported a significant correlation between the TPC of extracts of Jerusalem artichoke and their antioxidant activity using the DPPH assay, and a similar result was reported by Espinosa et al. [19] for extracts of orange peels using the ABTS assay. No correlations between antioxidant activity and concentration of phenolics or other compounds have been reported for SMS extracts.

Our results also revealed a positive correlation between the antioxidant activity and the concentration of caffeic acid (R^2^ = 0.81, PCC = 0.90, *p*-value < 0.05) (Figure 9b), indicating that caffeic acid is an essential contributor to the antioxidant activity observed in the SMS extracts. This agrees well with the existing knowledge on the antioxidant activity exerted by caffeic acid [36]. The low caffeic acid concentration of the UAE extracts (Table 4) might be behind their lower antioxidant activity compared to the other extracts (Figure 5c). Similarly, the higher antioxidant activity of SWE extracts (Figure 7c) might be linked to their higher caffeic acid concentration compared with that of the extracts obtained by other methods. On the other hand, the correlations found between the antioxidant activity and other phenolic acids had relatively low R^2^ values (Tables S7 and S8). Some correlation (R^2^ = 0.68, PCC = 0.82, *p*-value < 0.05) was observed between the antioxidant activity and the concentration of total carbohydrates (Table S9). That might be related to specific carbohydrates, such as β-glucans [37], which are typically contained in fungal fruitbodies and mycelium, and are known to show antioxidant activity [32,38].

### 2.6. Enzymatic Saccharification of SMS

**Figure 10:**
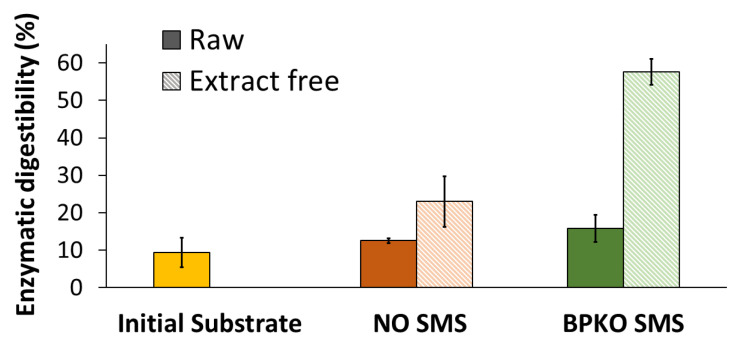
Enzymatic digestibility of cellulose contained in the initial substrate, raw and extract-free SMS of the BPKO and NO strains of *Pleurotus* spp. Mean values from triplicate experiments were used to build the graphs. The error bars show the standard deviations.

The enzymatic saccharification was evaluated by comparing the cellulose digestibility, i.e., the mass percentage of cellulose being saccharified, of different SMS samples upon treating them with cellulolytic enzymes. An analytical enzymatic saccharification assay [39] was applied. The initial substrate used for mushroom cultivation was also included in the experiment. The enzymatic digestibility of cellulose was 9.4% (*w*/*w*) for the initial substrate. In comparison, it was 12.5% and 15.8% for NO SMS and BPKO SMS, respectively (Figure 10). That corresponds to an enhancement of the enzymatic digestibility by 33% for NO SMS and 68% for BPKO SMS compared to the initial substrate. The enhanced enzymatic digestibility of cellulose in SMS compared with the initial lignocellulosic substrate is a consequence of fungal cultivation acting as a biological pretreatment [40]. While growing on lignocellulose, fungi remove part of lignin and hemicelluloses, and that improves the accessibility of cellulose to the enzymes. The digestibility pattern for both SMSs is in good agreement with their lignin content: BPKO SMS, which contained 16% lignin (Table 1), had better digestibility than NO SMS, which contained 18.4% lignin.

The observed enhancement of the enzymatic digestibility is higher than the previously reported by Zadrazil [41] for SMS of *P. ostreatus* grown on a wheat-straw substrate, but our absolute digestibility values were lower. However, the enzymatic digestibility achieved in the current work was below the expectations that one could have for a biologically pretreated material. It is expected that the delignification caused by fungal cultivation, i.e., a biological pretreatment, leads to an enhancement of the enzyme access to the substrate, which results in an improvement of the enzymatic saccharification [13]. The poor enzymatic digestibility observed in the current work might be attributed to pretreatment-related issues, such as the fungal strains or the cultivation conditions. It might be so that the cultivation time was not long enough to ensure an effective delignification. The lignin content in the SMS (16.0–18.4% (Table 1) was only slightly lower than in the initial substrate (19%, Table S1). The enzymatic digestibility values were lower than our previously reported results for *Lentinula edodes* SMS [12]. The lower enzymatic digestibility of the *Pleurotus* spp. SMS than that of *L. edodes* SMS is in line with the results of a previous study with the SMS of another oyster mushroom [42]. In that study, the enzymatic saccharification of SMS from the cultivation of *Pleurotus pulmonarius* on a birch-based substrate resulted in lower glucose yields than typical values for an *L. edodes* birch-based SMS. The result was attributed to the fact that the longer time required for shiitake cultivation provides a better pretreated SMS than that of oyster mushrooms, which requires less time for cultivation and results in a lower delignification degree.

The enzymatic saccharification was also assayed for the SMS after exhaustive extraction. For both SMSs, the enzymatic digestibility of cellulose was improved after the removal of the extractive compounds (Figure 10). For the NO SMS, the enzymatic digestibility increased approximately two-fold (from 12.5 to 23.0%), while for the BPKO SMS, an almost four-fold increase was observed (from 15.8 to 57.6%). The enhancement of the enzymatic digestibility after extraction can be attributed to the partial removal of extractive compounds that are inhibitory to the enzymes. It is known that certain phenolic compounds formed by lignin degradation or by solubilization of the extractive fraction of lignocellulose inhibit cellulolytic enzymes [43]. An investigation of the effect of SMS phenolic compounds on the inhibition of enzymatic saccharification is underway by this group.

Even if clear improvements were observed due to the extraction, the achieved digestibility values are still relatively low. The current study is a pioneering investigation on upgrading SMS by recovering bioactive compounds and producing sugars to be converted into high-added value products following a biorefinery approach. Developing an SMS-based biorefinery requires further improvement for producing enough sugars to back microbial fermentation processes of industrial relevance. A strategy to follow in that direction is to investigate how different extraction methods and operational conditions can improve enzymatic saccharification. Another strategy to consider is applying a post-treatment, e.g., with an acid or an alkali, to the extract-free SMS to improve its enzymatic digestibility further. It has been shown that treating the SMS of *Flammulina velutipes* with dilute sulfuric acid results in a high yield of reducing sugars [44]. It has also been reported that alkaline treatment of *Pleurotus florida* SMS facilitated the enzymatic saccharification to produce enough sugar for producing ethanol by yeast fermentation [45]. Furthermore, since the 5% (*w*/*w*) biomass load used in the enzymatic saccharification assay in this study is intended only for analytical purposes, larger loads are required for producing fermentable sugar amounts of industrial relevance. Therefore, preparative enzymatic saccharification protocols with biomass loads in the 10–20% range need to be developed and optimized for SMS from different sources.

## 3. Materials and Methods

### 3.1. Materials

SMS from the cultivation of two fungal strains was provided by Husbonden AS (Disenå, Norway). The cultivated fungi were a commercial strain, which is a *Pleurotus ostreatus* × *Pleurotus eryngii* hybrid, referred to as the Black Pearl King Oyster (BPKO) mushroom, and a wild *P. ostreatus* strain isolated in Norway, which is hereafter referred to as the Norwegian Oyster (NO) mushroom. The initial substrate (IS) used for mushroom cultivation was composed of 32% oak (*Quercur robur*) sawdust, 8% wheat (*Triticum aestivum*) bran, ~60% water, and 0.08% CaCO_3_. SMS and IS samples were dried at room temperature in a ventilation hood until a dry-matter (DM) content above 90% (*w*/*w*) was reached. The dry samples were shredded using a Robot Coupe shredder (Robot-Coupe SNC, Montceau-en-Bourgogne, France) and sieved to a particle size below 0.5 mm. The resulting powdered material was stored in sealable plastic bags until further use.

The chemicals were acquired from Sigma-Aldrich Chemie GmbH (Steinheim, Germany) if not stated otherwise. The enzyme blend Cellic CTec2, procured from Sigma-Aldrich, was used in the enzymatic saccharification experiments. It contains cellulases, β-glucosidases, other enzymes involved in cellulose degradation, and hemicellulolytic enzymes. The CMCase activity of the used enzyme preparation was 330 U/mL.

### 3.2. Characterization of Biomass Materials

The content of dry matter, mineral components (ash), extractive compounds, structural polysaccharides, and lignin in raw- and extract-free SMS and the initial substrate was determined using standard protocols. All the analyses were performed in triplicates, and mean values and standard deviations were reported.

Dry matter was determined gravimetrically by drying overnight at 105 °C as previously described [46]. A Termaks TS4057 oven (Termaks, Bergen, Norway) and a Sartorius analytical balance (Sartorius Lab Instruments GmbH, Göttingen, Germany) were used. For determining the content of mineral components, 2 g (dry weight (DW)) samples were incinerated at 575 °C based on the National Renewable Energy Laboratory (NREL) standard [47]. A Carbolite CWF 1100 muffle furnace (Carbolite Gero, Sheffield, UK) was used.

Extractive compounds were determined by sequential Soxhlet extraction using water and absolute ethanol according to NREL standard protocol [48]. Extraction was performed using 7 g of biomass and 250 mL of solvent at a 35:1 (*v*/*w*) solvent-to-biomass ratio (SBR). Water extraction was run for eight hours, and ethanol extraction was run for six hours. After extraction, the total volume of the liquid fraction was brought back to 250 mL, and vacuum filtration was performed using ashless filter paper (S & S, Dassel, Germany). Then, 10 mL samples were withdrawn and stored frozen for further analyses. After that, the solvent was recovered by vacuum evaporation using a Büchi R-114 rotary evaporator (BÜCHI Labortechnik AG, Flawil, Switzerland). The liquid remaining at the end of the operation was left to evaporate in pre-weighed beakers in a ventilation hood. Finally, the extracts were dried in an oven (Termaks) overnight at 35 °C for multiple days until constant weight.

The determination of structural carbohydrates and lignin was performed by analytical acid hydrolysis based on an NREL protocol [49]. Aliquots of 300 mg (DW) were mixed with 3 mL 72% sulfuric acid and hydrolyzed at 30 °C for 1 h in a water bath. Then, the samples were transferred to previously weighed 100 mL screw-top flasks, 84 mL distilled water was added to bring the concentration of H_2_SO_4_ down to 4%, and hydrolysis was run at 121 °C for 1 h in a CertoClav CV-EL 18L autoclave (CertoClav Sterilizer GmbH, Leonding, Austria). After that, the flasks with the hydrolysates were cooled to room temperature in an ice bath, and the weight was readjusted to the initial value by distilled water addition. The hydrolysates were then separated from the solid residue (Klason lignin) by filtering through pre-dried and pre-weighed filters. Klason lignin was determined gravimetrically after drying overnight at 105 °C in a Termaks oven. Then, 5 mL hydrolysate samples were stored frozen in plastic tubes for later analysis.

### 3.3. Extractions

#### 3.3.1. Soxhlet Extraction

A Soxhlet apparatus with an extractor chamber capacity of 200 mL was used with a heating mantle. Extractions with solvent systems containing water and absolute ethanol in 100:0, 80:20, 60:40, 50:50, 40:60, 20:80, and 0:100 (*v*/*v*) ratios were held for 4 h for the SMS of the BPKO strain. The SMS of the NO strain was extracted only with the 40:60 ethanol/water mixture. Eight grams (DW) of biomass samples were used, and the solvent was added at a volume ensuring an SBR of 25:1 (*v*/*w*). The extraction with 50:50 solvent mixtures was performed in triplicate, and the standard error of the experiment was calculated.

After all extractions, the liquid fractions containing the solvent and the extract were brought back to their starting volume by adding the corresponding solvent and then vacuum filtered as described above. Samples were taken to determine the extraction yield and extraction efficiency, and around 10 mL was stored frozen in Falcon tubes until further analyses. The remaining extract was concentrated by rotary evaporation (Büchi). For determining the EY, 2 mL aliquots of the liquid sample were dried overnight at 105 °C (Termaks), and the extract was weighed using an analytical balance (Sartorius). The EY was calculated as the percentage of extract from the mass of the SMS sample submitted to extraction. The EE was calculated as the mass percentage of the extract out of the total content of extractive compounds determined in the characterization. The concentrated extracts were frozen in 50 mL falcon tubes and used later to identify individual phenolic acids by HPLC. The extract-free solids were air-dried at room temperature. After that, their dry matter content was determined, and the rest was stored in plastic bags.

#### 3.3.2. Reflux Extraction

Reflux extraction was performed with a 250 mL round flask and a 250 mm reflux condenser together with a heating mantle, using a 40:60 (*v*/*v*) absolute ethanol/water mixture as a solvent system at the same solvent-to-biomass ratio as in the Soxhlet extraction. The extraction time was 60, 120, 150, 180, or 240 min. The extraction lasting 150 min was performed in triplicate. The extracts were processed following the same procedures used for Soxhlet extraction.

#### 3.3.3. Ultrasound-Assisted Extraction

UAE was performed with a 40:60 (*v*/*v*) ethanol/water mixture as a solvent system. A 25:1 (*w*/*v*) solvent-to-biomass ratio was used. A 2^3^ experimental design was applied, with the temperature (35, 50, and 65 °C) and extraction times (30, 45, and 60 min) as independent factors. Extraction was performed by immersing a sonication probe (Sonifier SFX250, Brandson, Mexico) in a 250 mL round wide-neck flask containing a suspension of the SMS in the solvent system. The ultrasonic frequency was 20 kHz, the maximum power was 250 W, and the amplitude was 50%. A cold-water bath was used to avoid overheating of the suspension. The extracts were separated and processed as described in previous sections.

#### 3.3.4. Subcritical Water Extraction

Subcritical water extraction was performed by heating a mixture containing 12 g (DW) of dry SMS suspended in 300 mL of distilled water in a pressurized Parr 4520 reactor (Parr Instrument Company, Moline, IL, USA). Two SWE regimes were applied. In one run, the SMS suspension was treated non-isothermally by heating it to 150 °C and cooling it to room temperature immediately afterward. In the second run, the temperature was held at 150 °C for 10 min before cooling to room temperature. The severity factor (log R_0_) was 1.8 in the first run and 2.3 in the second. The reactor was cooled by immersion in icy water.

### 3.4. Analytical Enzymatic Saccharification (AES)

Around 50 mg (DM) of raw and extract-free SMS was suspended in 900 µL of 50 mM sodium citrate buffer (pH 5.2) at a 5% (*w*/*w*) solids content in Eppendorf tubes. The tubes with the suspensions were mixed at 100 rpm for one hour in a Thermo Scientific compact digital waving rotator (Thermo Fischer Scientific, Waltham, MA, USA) placed in a Termaks B4115 incubator set at 45 °C. After that, 50 µL of a previously prepared stock solution of the cellulase blend Cellic CTec2 (Sigma-Aldrich, Steinheim, Germany) was added at a load of 80 CMCase units/g biomass. The reaction mixtures were then incubated under the same conditions for 72 h. Enzyme-free substrate controls and substrate-free enzyme blanks were run in parallel. After elapsing the reaction time, the resulting slurry was separated using a Heraeus Pico 21 centrifuge (Thermo Fisher Scientific, Osterode, Germany) at 20,200× *g* for 5 min at 4 °C. The supernatant, hereafter referred to as hydrolysate, was transferred to new Eppendorf tubes and stored frozen until further analysis, and the solid material was discarded. Glucose in the hydrolysates was determined by high-pressure liquid chromatography (HPLC). Glucose concentration was used for calculating the enzymatic digestibility. The enzymatic digestibility was calculated as the mass percentage of the cellulose contained in the AES assay saccharified to glucose.

### 3.5. Analytical Methods

#### 3.5.1. Total Carbohydrates

Determination of the concentration of total carbohydrates was performed based on a modification of the phenol–sulfuric acid protocol [50]. The method is based on reading the absorbance of a 1:3 (*v*/*v*) mixture of extract and concentrated sulfuric acid at 315 nm in a quartz cuvette using a UV 3100P spectrophotometer (VWR, Leuven, Belgium). The analysis was performed in triplicates.

#### 3.5.2. Total Phenolics

To determine the concentration of total phenolics, the Folin–Ciocalteau method [51] was used. The calibration standard was gallic acid dissolved in 10% ethanol. The absorbance was measured at 765 nm with a UV 3100P spectrophotometer (VWR). The analysis was performed in triplicates, expressing the results as gallic acid equivalents (GAE). The results were used for calculating the recovery of total phenolics in extractions from SMS samples.

#### 3.5.3. Antioxidant Activity

Antioxidant activity was determined essentially according to Guo et al. [52] using the ferric-ion reducing antioxidant power (FRAP) assay. The FRAP solution was freshly prepared by mixing 40 mM tripyridyltriazine (TPTZ) with 20 mM FeCl_3_ and acetate buffer (pH 3.6) in a ratio of 1:1:10. Distilled water (30 µL) was pipetted into each well of a Nunclon 96-well plate (Nalge Nunc International, Roskilde, Denmark) before adding 6 µL of sample, standard or blank (distilled water). The FRAP solution was preheated in a water bath at 37 °C for 10 min before adding 270 µL to each well. The microplate was incubated at 37 °C for 10 min before reading absorbance using a FluroStar Optima spectrophotometer (BMG Labtech, Offenburg, Germany) at 595 nm over 30 min. Sample measurements were performed in triplicates. TPTZ, FRAP solution, and gallic acid standards (0.01, 0.1, 0.25, 0.5, and 1.0 mM) were prepared fresh for every run.

#### 3.5.4. Ergosterol Determination

Ergosterol was extracted by mixing 0.5 g (DM) biomass with 15 mL ethanolic solution of KOH in a 30:1 (*v*/*w*) SBR. The mixture was held in a water bath at 85 °C for 1 h for saponification and then cooled to room temperature and vacuum filtered. In a separation funnel, ergosterol was recovered from the saponification mixture by two-fold liquid–liquid extraction with cyclohexane (Merck, Darmstadt, Germany). The resulting organic fractions from both extraction steps were pooled together. Quantification was performed by reading the absorbance at 285 nm with a UV 3100P spectrophotometer (VWR) and using analytical-grade ergosterol as the calibration standard.

#### 3.5.5. HPLC Determination of Sugars and Phenolic Acids

Glucose, xylose, and arabinose in the analytical acid hydrolysis, glucose in the enzymatic saccharification, and phenolic acids in the extract were determined by HPLC using an Ultimate 3000 (Dionex Softron, Germering, Germany) system. The separation of sugars in the analytical acid hydrolysates samples was completed with an Aminex HPX-87H column (Bio-Rad Laboratories, Hercules, CA, USA) held at 60 °C, and detection was performed with a Series 200 refractive index (RI) detector (Showa Denko, Tokyo, Japan). The mobile phase was a 5 mM sulfuric acid solution (VWR Chemicals, France), eluted at 0.6 mL/min flow for 40 min per sample. Glucose in the enzymatic saccharification was determined using a Rezex-RPM Monosaccharide Pb^+2^ column (Phenomenex, Torrance, CA, USA) heated to 85 °C and an RI detector (Showa Denko). The eluent was distilled water, filtered through a 0.45 µm membrane filter of regenerated cellulose (GE Healthcare, Buckinghamshire, United Kingdom), and eluted at 0.6 mL/min for 40 min per sample. The phenolic acids were separated on a Kinetex 5u C18 100A column (Phenomenex). A UV detector (Dionex Softron) at either 330 nm or 280 nm was used. HPLC was run at 25 °C with a flow of 1.5 mL/min for 18 min per sample. The mobile phase contained 95% of 0.1% trifluoroacetic acid (FLUKA Chemie AG, Buchs, Switzerland) and 5% acetonitrile (VWR Chemicals, France). HPLC-grade chemicals were used as standards. Before HPLC analysis, all the samples were appropriately diluted and filtered using 0.45 µm Nylon syringe filters (VWR, Radnor, PA, USA).

### 3.6. Statistical Analysis

The statistical significance was calculated using one-way and two-way ANOVA and Student’s *t*-test. Correlations between antioxidant activity and either concentration of total phenolic compounds or individual phenolic acids were determined using Microsoft Excel (version 2102), and the R^2^, Pearson’s correlation coefficient and *p*-value were calculated. Statistical analysis tables are provided as Supplementary Material (Tables S2–S9).

Microsoft Excel, Statgraphics Plus 5.0 for Windows (Manugistics Inc., Rockville, MD, USA), and MODDE 11.0 (Umetrics AB, Umeå, Sweden) were used for processing the results.

### 3.7. Formatting of Chemical Structures

Chemical structures were drawn using the molecular editing software ChemDoodle 10.3.0 (https://www.ichemlabs.com/, accessed on 10 April 2023).

## 4. Conclusions

This study provided basic knowledge on the suitability of different extraction methods for recovering bioactive compounds from spent mushroom substrate. The potential of ultrasound-assisted extraction and subcritical-water extraction for extracting bioactive compounds from the spent mushroom substrate of two *Pleurotus* spp. strains was shown. UAE allowed a comparable extraction yield under relatively milder conditions than reflux or Soxhlet extraction at higher temperatures and longer times. Among the four tested methods, SWE resulted in the highest extraction yield and efficiency, higher concentrations of phenolic compounds and total carbohydrates, and higher antioxidant activity of the extracts. The results of this study reveal clear directions about how the research ought to proceed for maximizing the recovery of bioactive compounds from SMS. Further experiments based on these findings are currently underway in our group.

A comparison of the extraction parameters for SMS of two fungal strains revealed a higher susceptibility of a wild *P. ostreatus* strain to yield extracts with higher antioxidant activity than that of a commercial *Pleurotus* spp. strain.

Several phenolic acids were identified in the extracts of SMS. The antioxidant activity of SMS extracts was found to be strongly correlated with their concentration of caffeic acid and total phenolic compounds.

Lastly, it was demonstrated that the enzymatic digestibility of cellulose contained in SMS is enhanced after the extraction of bioactive compounds.

## Figures and Tables

**Table 1:** Chemical composition of SMS from the cultivation of BPKO and NO strains, mass fractions in % (dry weight). Mean values from triplicate analyses are presented for all the components except extractives. Standard deviation is shown in parenthesis.

	BPKO	NO
Cellulose	38.3 (0.6)	37.5 (0.6)
Hemicelluloses	22.1	20.4
*Xylan*	18.5 (0.6)	16.6 (0.6)
*Anhydroarabinose*	3.6 (0.1)	3.8 (0.3)
Klason lignin	16.0 (0.6)	18.4 (0.9)
Water extractives	12.0	13.7
*Total carbohydrates*	10.7(1.0)	9.8 (0.3)
*Total phenolics*	1.0 (0.2)	1.0 (<0.1)
Ethanol extractives	1.5	1.3
*Total phenolics*	0.3 (<0.1)	0.2 (<0.1)
Total extractives	13.5	15.0
Ergosterol	<0.1	<0.1
Ash	2.0 (0.3)	2.7 (0.5)

**Table 2:** Experimental design used in the ultrasound-assisted extraction applied to BPKO SMS. Operational conditions of the design and extraction yields resulting from the experiment.

No.	Temperature, °C	Time, Min	Extraction Efficiency ^1^, g/100 g Biomass
1	35	30	10.9 (4.7)
2	35	45	20.1 (8.0)
3	35	60	12.4 (1.4)
4	50	30	61.6 (2.6)
5	50	45	60.7 (3.9)
6	50	60	67.5 (1.1)
7	65	30	69.4 (6.0)
8	65	45	80.8 (3.1)
9	65	60	81.7 (0.5)

^1^ Mean values from triplicate measurements. The standard deviations are shown in parentheses.

**Table 3:** Comparison of the results of different extraction methods applied to SMS of two *Pleurotus* spp. strains. Selected extraction conditions: Soxhlet with the E40 solvent mixture; E40 reflux for 120 min; E40 UAE at 65 °C for 60 min. Mean values from triplicate analyses. The standard deviations are shown in parentheses.

Parameter/Extraction Method	BPKO SMS	NO SMS
Extraction yield, % (*w*/*w*)		
*Soxhlet extraction*	9.5 (0.3)	10.7 (0.2)
*Reflux extraction*	8.5 (0.6)	12.6 (0.1)
*UAE*	11.1 (0.1)	12.3 (0.3)
Extraction efficiency, % (*w*/*w*)		
*Soxhlet extraction*	69.9 (1.9)	71.1 (1.5)
*Reflux extraction*	62.9 (4.6)	83.7 (0.5)
*UAE*	81.7 (0.5)	82.0 (2.2)
Total phenolics, mg GAE/g biomass		
*Soxhlet extraction*	9.1 (0.3)	8.3 (0.2)
*Reflux extraction*	8.6 (0.2)	9.5 (0.2)
*UAE*	5.6 (0.5)	7.4 (0.3)
Total carbohydrates, g/L		
*Soxhlet extraction*	7.8 (0.5)	6.1 (0.5)
*Reflux extraction*	8.1 (0.5)	7.9 (0.9)
*UAE*	7.3 (0.5)	7.4 (0.6)
Antioxidant activity, mM GAE		
*Soxhlet extraction*	0.4 (<0.1)	0.5 (<0.1)
*Reflux extraction*	0.4 (<0.1)	0.5 (<0.1)
*UAE*	0.3 (<0.1)	0.4 (<0.1)

**Table 4:** Concentration of phenolic acids ^1^ in selected extracts from BPKO SMS with different extractions methods, mg/L. Mean values from triplicate analyses. The standard deviations are shown in parentheses.

Extract Sample ^2^	Chlorogenic Acid	Caffeic Acid	*p*-Coumaric Acid	Ferulic Acid	Gallic Acid	Vanillic Acid
SoE-80	6.0 (0.2)	0.7 (0.2)	2.3 (0.1)	0.9 (0.1)	5.5 (0.4)	12.4 (1.0)
SoE-40	7.3 (0.3)	1.1 (0.2)	2.3 (0.1)	1.1 (0.1)	3.6 (0.4)	8.2 (2.5)
SoE-20	4.6 (0.3)	0.6 (<0.1)	1.6 (<0,1)	0.8 (<0.1)	3.0 (0.6)	6.9 (1.3)
ReE-60	4.8 (0.3)	0.7 (0.1)	1.7 (0.1)	0.7 (<0.1)	2.8 (0.3)	3.8 (0.4)
ReE-120	4.2 (0.2)	0.7 (0.1)	1.1 (0.1)	0.6 (<0.1)	2.8 (0.4)	4.5 (0.4)
ReE-240	4.8 (<0.1)	0.7 (0.1)	1.5 (0.1)	0.7 (0.1)	2.5 (0.3)	5.8 (0.6)
UAE 35–30	3.1 (<0.1)	0.2 (0.1)	1.3 (0.1)	0.5 (<0.1)	2.6 (0.1)	2.9 (0.3)
UAE 50–45	3.6 (<0.1)	0.3 (<0.1)	1.3 (0.1)	0.6 (<0.1)	3.2 (0.2)	1.8 (0.4)
UAE 65–60	4.6 (0.1)	0.3 (<0.1)	1.7 (<0.1)	0.8 (0.1)	3.0 (0.3)	3.0 (0.2)
SWE 10	5.4 (<0.1)	1.7 (0.4)	1.2 (0.1)	0.7 (0.1)	11.1 (0.5)	16.4 (0.2)

^1^ Determined by HPLC; ^2^ Sample codification: for Soxhlet extraction (SoE), the numeral indicates the volumetric share of ethanol in the solvent system; for reflux extraction (ReE), the numeral indicates the time (in min); for UAE, the numerals indicate, respectively, the temperature (in °C) and the time (in min); for SWE, the numeral indicate the holding time (in min).

## Data Availability

Data will be made available on request.

## Supplementary Materials

The following supporting information can be downloaded at: https://www.mdpi.com/article/10.3390/molecules28135140/s1, Table S1. Chemical composition of the initial substrate used for the cultivation of the BPKO and NO strains. Mass fractions in % (dry weight). Mean values from triplicate analyses are presented for all the components, except extractives. Standard deviation is shown in parenthesis. Table S2. One-factor ANOVA analysis with replication of total phenolic content in Soxhlet extractions. Based on triplicate measurements from nine extractions (E0-E100). Table S3. Two-factor ANOVA analysis with replication of total carbohydrate content in ultrasound-assisted extractions. Based on triplicate measurements from nine extractions at three extraction times and three temperatures (30, 45 and 60 min) (35, 50 and 65 °C). Table S4. One-factor ANOVA analysis with replication, of antioxidant activity (measured by FRAP) in reflux extractions. Based on triplicate measurements from seven extractions at different extraction times (60–240 min). Table S5. Correlation between caffeic acid concentration and antioxidant activity (FRAP). Table S6. Correlation between total phenolic content and antioxidant activity (FRAP). Data points from all extractions were included. Table S7. Correlation of ferulic acid concentration with antioxidant activity (FRAP). Table S8. Correlation of chlorogenic acid concentration with antioxidant activity (FRAP). Data points from all extractions were included. Table S9. Correlation between total carbohydrate content and antioxidant activity (FRAP). Data points from all extractions were included.
