# Supplementary material for: Evaluation of the Extraction of Bioactive Compounds and the Saccharification of Cellulose as a Route for the Valorization of Spent Mushroom Substrate
Source: Molecules. 2023 Jun 30;28(13):5140. doi: 10.3390/molecules28135140 (PMC10343705; doi:10.3390/molecules28135140)
Supplement: Supplementary file 1 [file molecules-28-05140-s001.zip › molecules-2453522-supplementary.pdf]

Evaluation of the extraction of bioactive compounds and the saccharification of cellulose as a route for the valorization of spent mushroom substrate

Sarah J. Klausen [1], Anne Bergljot Falck-Ytter [1], Knut Olav Strætkvern [1] and Carlos Martin [1,2,*]

[1] Inland Norway University of Applied Sciences, Department of Biotechnology, N-2317 Hamar, Norway; sa.schnell@gmail.com (S.J.K.); anne.falckytter@inn.no (A.B.F.-Y.); knut.stratkvern@inn.no (K.O.S.); carlos.medina@inn.no (C.M.)

[2] Umeå University, Department of Chemistry, SE-901 87 Umeå, Sweden

Supplementary materials

Table S1. Chemical composition of the initial substrate used for the cultivation of the BPKO and NO strains. Mass fractions in % (dry weight). Mean values from triplicate analyses are presented for all the components, except extractives. Standard deviation is shown in parenthesis.

	Initial substrate
Cellulose	36.1 (0.1)
Hemicelluloses	27.5
Klason lignin	18.8 (0.4)
Water extractives	7.2
Ethanol extractives	2.0
Total extractives	9.4
Ash	2.0 (<0.1)

Table S2. One-factor ANOVA analysis with replication of total phenolic content in Soxhlet extractions. Based on triplicate measurements from nine extractions (E0-E100).

Source of Variation	*Sum of Squares*	*Degree of freedom*	*Mean Square*	*Fishers-statistic*	*P-value*	*F critical*
Between Groups	*232349.3*	*8*	*29043.66*	*420.5824*	*7.42E-19*	*2.510158*
Within Groups	*1243.005*	*18*	*69.05583*			
Total	*233592.3*	*26*				

Table S3. Two-factor ANOVA analysis with replication of total carbohydrate content in ultrasound-assisted extractions. Based on triplicate measurements from nine extractions at three extraction times and three temperatures (30, 45 and 60 min) (35, 50 and 65 ˚C).

Source of Variation	*Sum of Squares*	*Degree of freedom*	*Mean Square*	*Fishers-statistic*	*P-value*	*F critical*
Sample (Temperature)	20.91902	2	10.45951	56.08168	1.849E-08	3.554557
Columns (Time)	0.295382	2	0.147691	0.791889	0.4681492	3.554557
Interaction	2.19708	4	0.54927	2.945069	0.0490992	2.927744
Within	3.357089	18	0.186505			
Total	26.76857	26				

Table S4. One-factor ANOVA analysis with replication, of antioxidant activity (measured by FRAP) in reflux extractions. Based on triplicate measurements from seven extractions at different extraction times (60-240 min).

Source of Variation	*Sum of Squares*	*Degree of freedom*	*Mean Square*	*Fishers-statistic*	*P-value*	*F critical*
Between Groups	0.03082	6	0.005137	2.59E+30	1.7E-209	2.847726
Within Groups	2.77E-32	14	1.98E-33			
Total	0.03082	20				

Table S5. Correlation between caffeic acid concentration and antioxidant activity (FRAP).

**Coefficient (r):**	0.90037366
**Number of data points:**	10
**T statistics:**	5.85276631
**Degree of Freedom:**	8
***p*-value:**	3.81580E-04

Table S6. Correlation between total phenolic content and antioxidant activity (FRAP). Data points from all extractions were included.

**Coefficient (r):**	0.89512921
**Number of data points:**	23
**T statistics:**	9.20128968
**Degree of Freedom:**	21
***p*-value:**	8.15234E-09

Table S7. Correlation of ferulic acid concentration with antioxidant activity (FRAP).

**Coefficient (r):**	0.3946195
**Number of data points:**	10
**T statistics:**	1.21473476
**Degree of Freedom:**	8
***p*-value:**	2.59102E-01

Table S8. Correlation of chlorogenic acid concentration with antioxidant activity (FRAP). Data points from all extractions were included.

**Coefficient (r):**	0.59772873
**Number of data points:**	10
**T statistics:**	2.10881309
**Degree of Freedom:**	8
***p*-value:**	6.79988E-02

Table S9. Correlation between total carbohydrate content and antioxidant activity (FRAP). Data points from all extractions were included.

**Coefficient (r):**	0.82717863
**Number of data points:**	23
**T statistics:**	6.74558663
**Degree of Freedom:**	21
***p*-value:**	1.13029E-06
